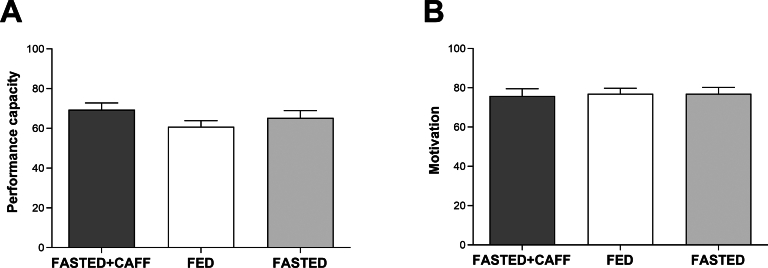# Carbohydrate Restriction During Recovery from High-Intensity–Interval Training Enhances Fat Oxidation During Subsequent Exercise and Does Not Compromise Performance When Combined With Caffeine

**DOI:** 10.1016/j.cdnut.2024.104520

**Published:** 2024-12-10

**Authors:** Camilla Soegaard, Simon Riis, Jesper Friis Mortensen, Mette Hansen

**Affiliations:** Department for Public Health, Aarhus University, Aarhus, Denmark

**Keywords:** periodized carbohydrate intake, moderately trained women, fat oxidation, caffeine, exercise performance

## Abstract

**Background:**

Carbohydrate restriction can alter substrate utilization and potentially impair endurance performance in female athletes. Caffeine intake may mitigate this performance decrements.

**Objectives:**

The aim of this study was to test the hypothesis that maximal fat oxidation (MFO) rate would be enhanced in the carbohydrate (CHO) restricted state in trained females. Additionally, the impact of caffeine intake before exercise under conditions of low CHO availability was examined on time-trial performance.

**Methods:**

By using a randomized, double-blinded, placebo-controlled, crossover design, 17 female endurance athletes completed 3 experimental blocks. Each block consisted of high-intensity-interval–training (HIT) in the evening, followed by a fat oxidation test to measure MFO rate and a 20-min time trial (20TT) performance the next morning. The females received standardized, isoenergetic diets with different timing of CHO intake: No CHO between exercise sessions without (FASTED) or with 300 mg caffeine (4.1–4.9 mg/kg body mass) (FASTED+CAFF) before morning exercise tests or CHO ingestion after HIT (FED).

**Results:**

MFO rate was higher in FASTED+CAFF (0.57 ± 0.04 g/min) than that in FED (0.50 ± 0.04 g/min, *P* = 0.039) but not different from FASTED condition. Power output performed during the 20TT was higher after FASTED+CAFF (189 ± 9 W) than that after FASTED (+6.9%, *P* = 0.022) and FED (+4.2%, *P* = 0.054).

**Conclusions:**

CHO restriction during recovery from HIT enhances MFO rate during subsequent exercise compared with the condition where CHOs were consumed during the recovery period, but the effect was only significant when CHO restriction was combined with caffeine supplementation before the MFO test. In addition, caffeine ingestion before exercise in the CHO-restricted state compensates for the decreased work capacity associated with the CHO-restricted state.

## Introduction

Prolonged endurance performance highly depends on skeletal muscle oxidative capacity and the availability of exogenous and endogenous carbohydrate (CHO) [1]. Given that glycogen stores are relatively small and act as a limiting factor for endurance performance, various training strategies involving periodic CHO restriction have been explored to enhance fat oxidative capacity of the skeletal muscle during training [2,3] and, consequently, improve performance without compromising the capacity to use glycogen. This includes training twice a day with minimal CHO intake in-between, training after an overnight fast, refraining from CHO in the hours following an exercise bout or combinations of these strategies [4]. The majority of studies have reported that chronic or periodic train-low augments the response to exercise in muscle cell signaling and gene expression, training-induced increases in oxidative enzyme activity/protein content and increases fat oxidation during submaximal exercise [3]. In untrained or moderately trained athletes, periodic CHO restriction during training have led to both superior adaptations and performance benefits in several [[5], [6], [7], [8]] but not all studies [9,10]. By contrast, performance improvements in endurance-trained male athletes have generally been lacking or comparable with improvements in controls training with high CHO availability [8,[10], [11], [12], [13], [14]]. The lack of improvements in well-trained endurance athletes could likely be related to the difficulty in reducing muscle glycogen sufficiently to gain superior effects [14] potentially due to initial larger glycogen stores [1] and/or an enhanced capacity to produce glucose via gluconeogenesis [15]. Furthermore, the fat oxidative capacity may already be very high in endurance-trained cohorts and not a limiting factor for endurance performance. A third explanation may be related to the reduced training quality when endurance exercise training is performed under CHO-restricted conditions [11,12].

One way to improve training quality with low CHO availability is to ingest caffeine before endurance exercise [16]. Hence, a low-dose caffeine ingestion [3 mg/kg body mass (BM)] increased power output significantly (3.5%) independently of muscle glycogen concentration in endurance-trained male cyclists [17]. However, to our knowledge, no studies have investigated how CHO restriction with or without caffeine supplementation influences performance and fat oxidation during exercise in female athletes [18]. Therefore, moderately endurance-trained female athletes were included to investigate the effect of CHO restriction after a high-intensity–interval training (HIT) session in the evening (sleep-low) on maximal fat oxidation (MFO) rate and endurance performance in a 20-min time trial (20TT) in the morning. The hypothesis was that MFO rate would be enhanced in the CHO-restricted state. An additional hypothesize was that caffeine supplementation before the test session in the morning would compensate for performance impairment in 20TT in the CHO-restricted situation.

## Methods

### Subjects

Twenty-four endurance-trained, healthy, nonsmoking, eumenorrheic females volunteered to participate in the study. They had a history of ≥3 y of competitive cycling (mountain biking, road cycling, or triathlon), and their mean training volume over the last 3–6 mo was 9–10 h/week consisting of a combination of cycling, running, swimming and strength training. The inclusion criteria were as follows: the females should be aged 18–40 y, have a maximal oxygen uptake ($\dot{V}$O_2max_) of ≥45 mL/min/kg, and have a regular menstrual cycle with ovulation the last 3 mo (cycle length between 22 and 35 d). Females reporting that they were eating a CHO-restrictive diet or practiced time-restricted diets were not included. Moreover, during the screening procedure, we asked for any history of eating disorders or weight instability within the last year (±>5 kg), which were exclusion criteria. Users of oral contraceptives were informed to continue to use the same type of oral contraceptives during the experimental period, whereas nonusers were informed to continue not to use hormonal contraception.

Owing to no-positive ovulation test, 2 subjects not using hormonal contraception were excluded before the study started. Five dropped out because of a knee injury, illness, and personal reasons. The analyses are therefore based on the 17 subjects who completed all 3 test blocks within 3–5 mo (Table 1). The participants were weight stable throughout the study period: trial 1, 62.0 ± 1.4 kg; trial 2, 62.1 ± 1.3 kg; and trial 3, 61.9 ± 1.4 kg (*P* = 0.464), and did not change in body FM: trial 1, 25.6% ± 1.1%; trial 2, 25.7% ± 1.0%; and trial 3, 25.5% ± 1.1% (*P* = 0.122).

**TABLE 1:** Subject characteristics.

Variable	Women (*N* = 17)
Age (y)	26 ± 4 (18–32)
Body mass (kg)	62 ± 6 (54–73)
Height (m)	1.68 ± 0.05 (1.59–1.78)
BMI (kg/m^2^)	22 ± 2 (18.5–25.9)
PPO (W)	279 ± 28 (243.0–343.3)
PPO (W/kg)	4.5 ± 0.5 (3.8–5.7)
HR_max_ (beats/min)	190 ± 10 (170–210)
$\dot{V}$O_2max_ (mL/min)	3.2 ± 0.4 (2.6–4.0)
$\dot{V}$O_2max_ (mL/kg/min)	52.2 ± 4.8 (45.4–61.0)
20-min TT at baseline (W)	209 ± 25 (173–269)

Values are means ± SD (range).

*Abbreviations:* HRmax, maximal heart rate; PPO, peak power output; $\dot{V}$O_2_max, maximal oxygen uptake; TT, time trial.

All subjects were informed of the purpose of the study and the possible risks before they gave their written consent to participate. The study protocol was approved by The Research Ethics Committees of Region Midtjylland, Denmark (journal no. 1-10-72-121-16).

### Study overview

**FIGURE 1:**
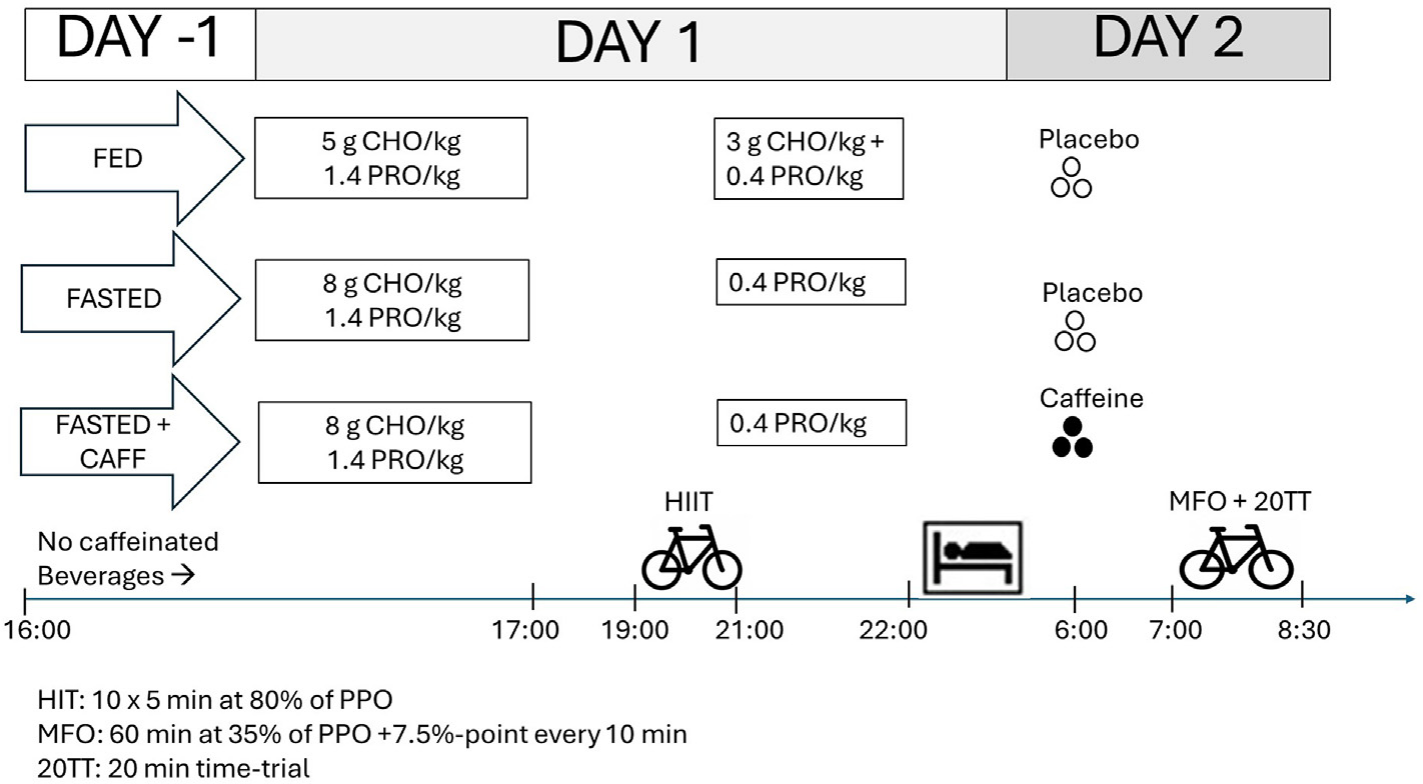
Study overview showing the 3 experimental blocks conducted in randomly assigned order by all 17 subjects. Blood, blood glucose and lactate measurement; CAFF, caffeine; CHO, carbohydrate; HIT, high-intensity-interval training session; MFO, maximal fat oxidation test; PPO, peak power output; PRO, protein; 20TT, 20-min time trial.

The study was designed as a randomly assigned, crossover, placebo-controlled study with 3 experimental blocks of each 2 d. Subjects performed 2 exercise sessions in each experimental block: the first exercise session, an HIT, was undertaken in the evening of the first experimental day, and the second exercise session, an MFO rate test followed by a 20TT, was performed the following morning on the second experimental day. On the HIT day, the subjects received a standardized, isoenergetic diet. Only the timing of the dietary CHO intake (total 8 g CHO/kg) differed between the 3 experimental blocks to manipulate the CHO availability when performing the MFO rate test and 20TT. In 1 block, the subjects received CHO before (5 g CHO/kg) and after (3 g CHO/kg) the HIT and before going to sleep (FED), whereas in the 2 other blocks, all CHOs were ingested during the day before the HIT, whereas CHO intake after HIT was prohibited (FASTED). The following day, the subjects performed the MFO rate and 20TT test in the overnight fasted state. One hour before the tests, the subjects ingested either 300 mg caffeine [4.1–4.8 mg/kg in the FASTED state (FASTED+CAFF) or placebo in the 2 other blocks (FED and FASTED)]. A person not related to the project randomly assigned the order of the 3 experimental blocks and handed out the pills (placebo or caffeine). The test personnel and participants were aware of the timing of the CHO intake, whereas both the test personnel and the participants were blinded regarding the intake of pills (placebo or caffeine). An overview of the study design is depicted in Figure 1.

### Preliminary testing

#### Maximal oxygen consumption and peak power output

Body composition [BM and fat mass (FM)] was determined using a Tanita body composition analyzer (SC-331S Total Body Composition Analyzer). $\dot{V}$O_2max_ and peak power output (PPO) were determined before subjects’ first experimental block by an incremental cycling test at a self-selected cadence to exhausting on a stationary bicycle ergometer (SRM). The height of the saddle and handlebar was set to match the usual position at the subjects’ own bike and were standardized before all exercise sessions. The cycle ergometer was equipped with individual racing pedals and toe clips, allowing subjects to wear their own shoes. After a 10-min warmup at the subjects’ own choice, the $\dot{V}$O_2max_ test started and stayed at a fixed workload (2 W/kg) for 2½ min, with an increase of 50 W the next 2½ min. The test continued to increase 25 W every 2½ min until volitional exhaustion [19,20]. Verbal encouragement was given to the subjects near the end of the test, but otherwise, the subjects did not get any auditory or visual feedback on performance except cadence. Heart rate (HR) (Polar m400), PPO and oxygen and carbon dioxide consumption ($\dot{V}$O_2_ and $\dot{V}$CO_2_) were recorded continuously throughout the test (Oxigraf O2CPX). The test stopped when the subjects dropped 10 revolutions per minutes (RPM) below their chosen cadence and exceeded *R* = 1.15.

$\dot{V}$O_2max_ was defined as the mean of the 2 highest successive $\dot{V}$O_2_ values of 15 seconds the subject achieved during the $\dot{V}$O_2max_ test. PPO was calculated as follows: PPO = *W* completed + (t/150 ×25), where *W* is the workload in watts during the last fully completed workload step, and *t* is the number of seconds in the final uncompleted workload step [20]. The individual’s PPO was used to calculate the workload intensities (in watts) during the HIT and MFO rate test.

#### 20TT familiarization

All subjects completed a 20TT familiarization trial on the SRM bike. They were asked to perform the highest mean power output possible during the 20 min. The SRM was programmed in the mode open end test and was fixed in gear 9 throughout the trial. In this mode, power output could change with pedal rate and on the subjects’ request from workload 1 to workload 10. For each 20TT time, distance and power were recorded every second by the SRM software. To avoid any experimental bias, the only feedback the subjects got was time elapsed and cadence. In addition, the subjects were verbally encouraged in the last 5 min. Intake of food and caloric fluids were prohibited during the testing although water was available ad libitum.

### Standardization of diet, training, and menstrual cycle

The diet protocol in this trial was inspired by that reported in Lane et al [21], who performed a study in male athletes to investigate effects of sleeping with reduced CHO availability on acute training responses. Similar to that reported by Lane et al. [21], the female participants on the HIT day followed a standardized, isoenergetic diet and fluid plan (containing 8 g CHO/kg, 1.8 g protein/kg, 1.5 g fat/kg, and 2 L water) with different timing of CHO intake to ensure divergent periodization over each trial according to the allocated experimental block (Figure 1). Similar to the protocol by Lane et al. [21], we chose to provide an isocaloric diet with similar CHO content in all experimental blocks, since we aimed to investigate effects of periodization of CHO intake around training sessions.

All food in the diet plan was provided to the participants, together with a diet plan. During 2 experimental blocks (FASTED and FASTED+CAFF), subjects consumed 8 g CHO/kg, 1.4 g protein/kg and 1.5 g fat/kg throughout the day with the last meal consumed before 17:00. Immediately after the HIT session, they got the remaining 0.4 g protein/kg as powder (Lacprodan SP-9225 Instant Whey Protein Isolate; Arla Foods Ingredients Group P/S) dissolved in 0.5 L water together with 2 × 250-mg sodium tablets (sodium chloride medic 250 mg; Meda AS) to preserve muscle protein synthesis, stimulate fluid retention, and thereby reduce risk of toilet visits during the night. In addition, they were provided with 0.5 L of water to drink over the night. During the experimental block FED, subjects consumed 5 g CHO/kg, 1.4 g protein/kg, and 1.5 g fat/kg before 17:00 with the remainder of the day’s intake (3 g CHO/kg and 0.4 g protein/kg) consumed after the HIT session. The diet plan was assembled of varied, healthy, Scandinavian food (breakfast: oats, milk, fruits, almonds, raisins; snacks: fruit; lunch: bread, cheese, ham, vegetables; dinner: pasta, tomato sauce, vegetables, chicken, and olive oil; after HIT: protein beverage as described earlier). The FASTED and FED diet plan differed by the timing of the intake of dried fruit and dextrose (corresponding to in total 3 g CHO/kg), which was consumed as a snack during day 1 in the FASTED condition and after the HIT in the FED condition.

In all 3 experimental blocks, the subjects slept at home. When they arrived in the laboratory the following morning, they ingested 0.3 L of water together with either 3 × 100-mg caffeine pills (Skanderborg Pharmacy, Skanderborg, Denmark) (FASTED+CAFF) or 3 placebo pills each consisting of 10 μg vitamin D (Natur Drogeriet D-vitamin; FASTED and FED) 1 h before initiating the MFO rate test. The placebo pills had the same number, size, and color as the caffeine pills to ensure that both subjects and test personal were blinded. No breakfast was eaten before the tests started on day 2. In all blocks, the food and beverages were consumed onsite immediately after the HIT session in the presence of the test personnel. The participants were asked before the HIT session and again before the MFO rate test if there had been any deviations from the diet plan. Only very minor deviations were reported. Therefore, the compliance to the diet protocol was rated to be very high. The subjects were instructed to avoid any strenuous physical activities 24 h before the HIT day as well as caffeine drinks after 16:00 the day before the HIT day. Habitual users of caffeine may experience side effects when suddenly refraining from consuming caffeine, but potential side effects in relation to the HIT session have likely been similar between blocks. Nevertheless, we cannot rule out that 20TT performance in the FED and FASTED state (without caffeine) may have been negatively impacted by withdrawal of caffeine in habitual users of caffeine/coffee consumers.

They were also asked to maintain a similar training level and volume throughout the course of the study period to minimize variations in training status. Before each experimental block, the participants were asked if they had been injured, sick or for other reasons been hindered to maintain their training schedule. Furthermore, they were asked if they had made any major change in their training habits, which could have impacted on their physical fitness level.

To account for a possible influence of menstrual/pill cycle on the outcome parameters, the experimental blocks were performed in the luteal phase of the menstrual cycle 5–9 days after a positive ovulation kit test (Babyplan Ovulation Test) in nonusers of hormonal contraception and between the 19^th^ and 23rd day of the pill cycle in users of oral contraceptives.

### The HIT session

After following the diet and fluid plan throughout the first day of each experimental block, the subjects performed the HIT session on the SRM bike in the evening at 19:00–21:00 with only 1 h of intraindividual variance to eliminate circadian variation in metabolism and performance [22]. After a standardized warmup (8 min at 60% PPO, 1 min at 100 W, and 1 min at 60% PPO), the subjects undertook the HIT, which consisted of 10 × 5-min workout bouts at 80% of the individual PPO with 2 min active recovery (100 W) between the work bouts. If the subjects had problems with completing the HIT as described, first step was to give an additional 1-min break between intervals. The second step was to give an additional 2-min break, and third step was to decrease the workload with maximal 50 W to allow the subjects to complete the remaining work bouts. If the workload was decreased in 1 HIT session, the same procedure was repeated for the next HIT sessions. HR was recorded from start to finish of the HIT session. $\dot{V}$O_2_ and $\dot{V}$CO_2_ was determined from the last minute of the third recovery period and during the next 4 work intervals including the recovery periods until the end of the first minute of the seventh recovery period. Ratings of perceived exertion (RPE) were recorded at the last minute of every work interval; 0.1 L of water was given in the recovery period after the warmup and in the recovery period between the work intervals.

### MFO rate test

In the morning of the second day of each experimental block, the subjects’ BM and FM were determined using the Tanita body analyzer. Thereafter, the subjects were asked to rate their motivation for the following test and sense of performance capacity as earlier described [19] before completing the standardized MFO rate test on the SRM bike with fixed work rates. The test took place at 06:00-09:00 with only 1 h of intraindividual variance between experimental blocks. Blood glucose (HemoCue Glucose 201 RT; HemoCue AB) and lactate concentration (Scout+ and sensors code 75; SensLab GmbH) were measured 5 min before test start. The MFO rate test started at 35% of the individual PPO increasing 7.5% PPO every 10 min for 60 min. HR was recorded continuously throughout the test, whereas $\dot{V}$O_2_ and $\dot{V}$CO_2_ were measured in the last 3 min of every step with a mouthpiece and a nose clip. Blood glucose and lactate were measured at minute 8 of every step from a blood sample taken from the fingertip, whereas RPE was recorded at the end of each step.

Whole-body rates of MFO rate were calculated from the respiratory data collected during MFO rate test (the last 80 s of intervals). The calculations were made from $\dot{V}$CO_2_ and $\dot{V}$O_2_ measurements under the assumption that protein oxidation was negligible [23]: fat oxidation = 1.695 × $\dot{V}$O_2_ − 1.701 × $\dot{V}$CO_2_, where fat oxidation rate given in grams per minute and $\dot{V}$O_2_ and $\dot{V}$CO_2_ in liters per minute.

Determination of MFO rate and the intensity at which MFO rate was obtained (Fat_max_) were determined according to previously described methods [24]. After each MFO rate test, a third-degree polynomial regression analysis was performed using fat oxidation rates as a function of measured intensity (%*V*O_2max_).

### 20TT test

Following the MFO rate test, the subjects had a 5-min break before the 20TT started where they were instructed to perform as high power output as possible. The only feedback the subjects got was time elapsed and cadence until the last 5 min of the 20TT where the subjects were verbally encouraged.

### Statistical analysis

All data were checked for sphericity and normality by Shapiro–Wilk test, histogram, and normal Q–Q Plot (SPSS, version 10.0). The following statistical analyses were conducted in GraphPad Prism (version 6.07). To determine differences in fat oxidation rates, respiratory exchange ratio (RER), HR, PRE, and lactate and blood glucose concentrations during the MFO rate test between experimental blocks, data were analyzed by a 2-way analysis of variance (ANOVA) with repeated measures (experimental block × intensity/time). When the statistical analysis showed a significant main effect and/or interaction, a Tukey multiple comparison test was used to identify pairwise differences between mean values. Likewise, to determine differences in PRE and HR during the HIT session between the 3 experimental blocks, data were analyzed using a 2-way ANOVA with repeated measures. To determine differences in 20TT, MFO rate, FAT_max_, motivation, and sense of performance capacity between experimental blocks, a 1-way ANOVA was used. When the results from the statistical analysis showed a *P* value of <0.10, a paired student *t* test was used as post hoc test. A paired samples *t* test was conducted to compare MFO rate and Fat_max_ for the 3 experimental conditions. Effect sizes were calculated using Cohen *d* for paired samples, which was determined by dividing the mean of the difference scores by the standard deviation of the difference scores. Cohen *d* was interpreted based on standard thresholds, where *d* = 0.2 represents a small effect, *d* = 0.5 a medium effect, and *d* = 0.8 a large effect. Analyses for trial order effect of MFO rate, 20TT, BM, FM, motivation, and sense of performance capacity were conducted by using a 1-way ANOVA. For all statistical analyses, significance was set at *P* < 0.05, and *P* < 0.10 was considered as a tendency. All data are expressed as mean ± SEM with 17 subjects unless otherwise stated.

#### Missing data points

All HR (% of max) data points for 2 subjects in the MFO rate test (FASTED+CAFF and FASTED, respectively) were replaced with their own means from the other 2 trials. All missing lactate data points in FED for 1 subject were replaced with her own means from the other 2 trials. Likewise, all missing lactate values in FASTED+CAFF from another subject were replaced with her own means from the other 2 trials.

#### Sample size calculation

To our knowledge, no other studies have used a similar 20TT test as in this study to test the effect of CHO restriction and caffeine supplementation in young, trained females. Hence, a previous study investigated the effect of a caffeine supplement at a 10-km time trial in a group of 8 endurance-trained males and one endurance-trained female (27.4 ± 5.9 y and 57.5 ± 3.9 mL/kg/min) with normal glycogen stores [25]. The distance in the latter trial is comparable with the distance the participants reached in the 20TT in this study (20TT: 9.3 ± 0.3 km, *n* = 17). A sample size calculation, based on findings from Astorino et al. [25] that reported a 1.9% improvement in the 10-km time-trial following caffeine supplementation, indicated that a minimum of 13 subjects would be required for the crossover study to achieve a power of 80% to detect a significant effect of caffeine supplementation. This calculation assumed a 5% significance level (1 sided) and SD of 0.44% [26]. The sample size calculations were based on the ergogenic effect of caffeine supplementation observed in a study where the participants were not CHO restricted, but caffeine supplementation has previously been observed to increase power output in males independently of muscle glycogen content [17]. Additionally, Lane et al. [17] observed a greater effect of caffeine with low (3.5%) compared with normal glycogen availability (2.8%), indicating that the calculated sample size for this trial was a conservative estimated.

## Results

### Whole-body fat oxidation rate

**FIGURE 2:**
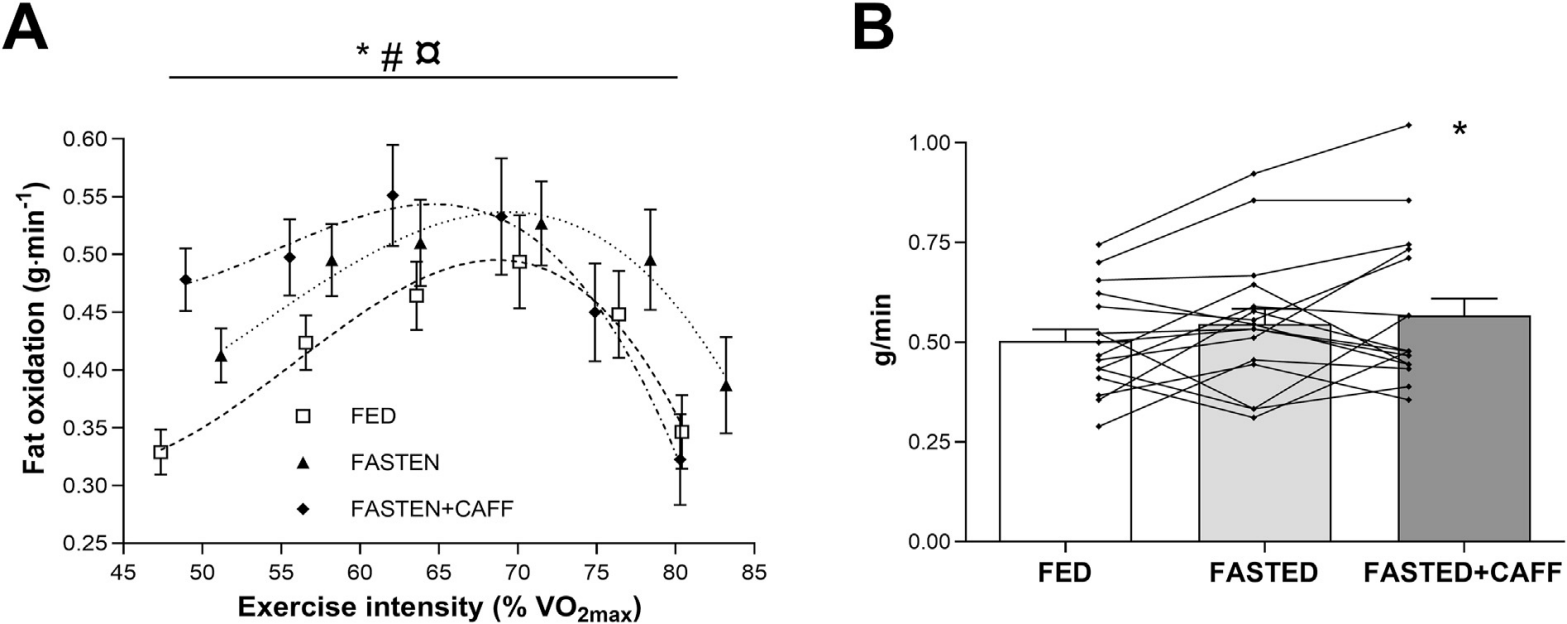
Fat oxidation rates (A) and maximal fat oxidation (MFO) rate (B) during the submaximal testing in the 3 different experimental blocks. (B) Lines indicate individual MFO rate. Data are shown as mean ± SEM; *n* = 16. (A) ∗FASTED+CAFF > FED, *P* = 0.087; #FASTED > FED, *P* = 0.106; ¤FASTED+CAFF > FASTED, *P* = 0.994. (B) ∗FASTED+CAFF > FED, *P* = 0.039. FASTED, no carbohydrate between exercise sessions; FASTED+CAFF, no carbohydrate between exercise sessions, but caffeine before exercise; FED, carbohydrate between exercise sessions.

The relationship between fat oxidation rates (grams per minute) and exercise intensity (% $\dot{V}$O_2max_) during the MFO rate test within each experimental block is presented in Figure 2A. The changes in fat oxidation during the test differed significantly between experimental blocks (experimental block × time *P* < 0.001) especially at the low intensity steps. Already, at the first data point of the test, fat oxidation was significantly higher in FASTED+CAFF than that in FASTED (*P* < 0.05) and FED (*P* < 0.001). Likewise, fat oxidation was higher in FASTED than FED (*P* < 0.001) in the initial phase of the test. However, although the fat oxidation curve in FASTED shifted upward compared with FED, this did not reach statistical significance (FASTED compared FED, *P* = 0.106; FASTED+CAFF compared with FED; *P* = 0.087).

MFO rate was significantly higher in FASTED+CAFF (0.57 ± 0.04 g/min; range: 0.37–0.99) than that in FED (0.50 ± 0.04 g/min; range: 0.31–0.72, *P* = 0.039, effect size 0.42) but not than that in FASTED (0.55 ± 0.04 g/min; range: 0.33–0.88, *P* = 0.473, effect size 0.12) (Figure 2B). Fat_max_ tended to be lower in FASTED+CAFF (65.8 ± 1.5% *V*O_2max_; range: 53.2–75.7) than that in both FASTED (69.3 ± 1.7% *V*O_2max_; range: 56.8–85.5; *P* = 0.067, effect size 0.53) and FED (68.7% ± 1.7% *V*O_2max_; range: 56.1–79.7; *P* = 0.127, effect size 0.44) (Figure 2A).

### RER, RPE, and HR

**FIGURE 3:**
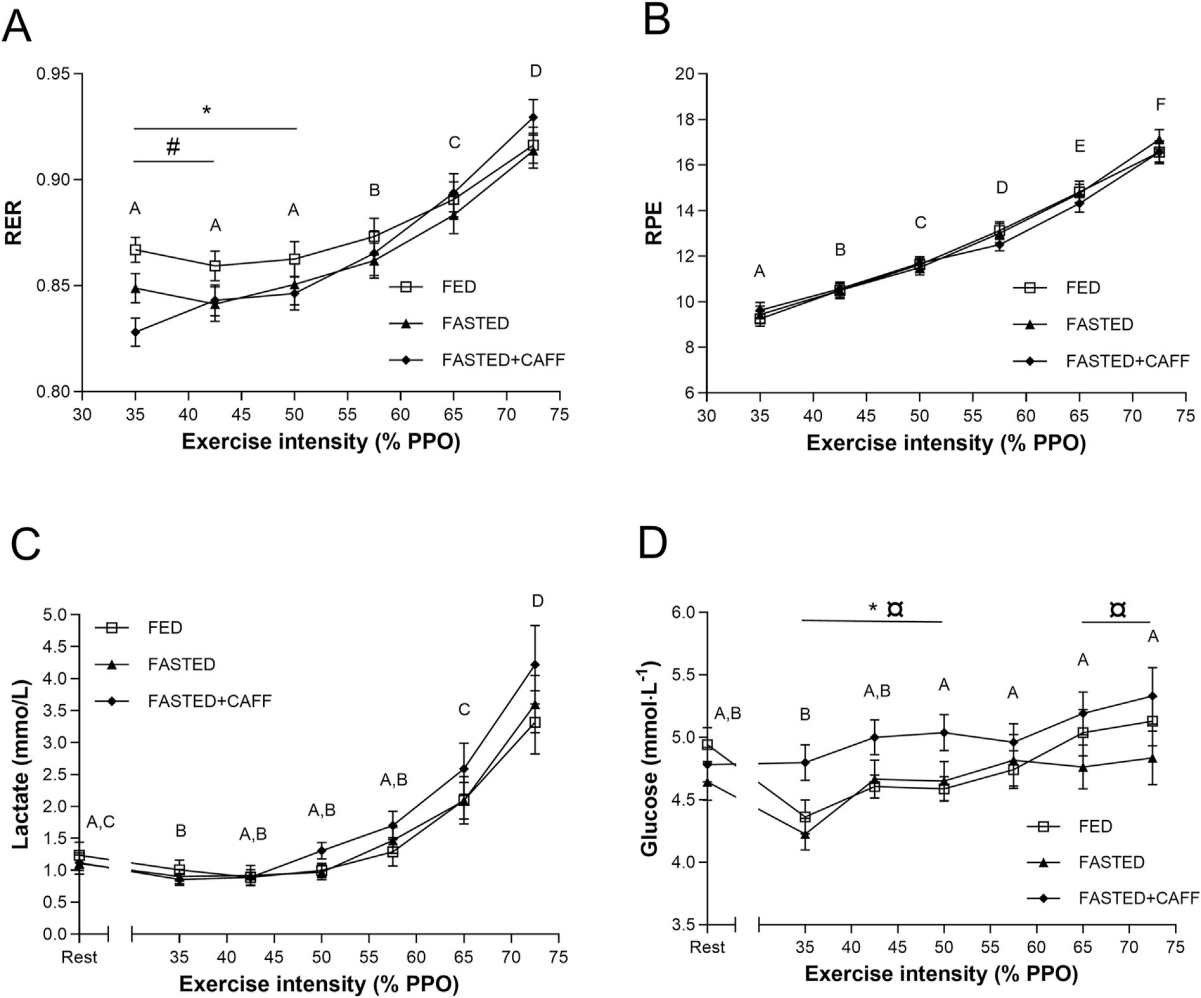
Respiratory exchange ratio (A), ratings of perceived exertion (B), lactate (C), and blood glucose (D) during the maximal fat oxidation (MFO) rate test (*n* = 16). Different letters indicates significant difference in the parameters between intensity steps (*P* < 0.05). (A) Experimental block × intensity interaction, *P* < 0.001; #FED > FASTED (*P* < 0.05); ∗FED > FASTED+CAFF (*P* < 0.05). (D) Experimental block × and intensity, *P* < 0.040; ∗FED < FASTED+CAFF (*P* < 0.05);, ¤FASTED+CAFF > FASTED, *P* < 0.05. FASTED, no carbohydrate between exercise sessions; FASTED+CAFF, no carbohydrate between exercise sessions, but caffeine before exercise; FED, carbohydrate between exercise sessions.

RER increased significantly during the MFO rate test (*P* < 0.001) and was significantly enhanced compared with baseline after the fourth interval and kept increasing thereafter until the end of the test (Figure 3). Furthermore, the 2-way ANOVA of the RER data showed a significant interaction (experimental block × time, *P* < 0.001). Post hoc tests showed that RER was higher during FED the first 3 steps of the MFO rate test than that during FASTED+CAFF and compared with the first 2 steps with FASTED. During the last 3 intensity steps of the MFO rate test RER did not differ significantly between the 3 experimental blocks. RER was only higher in FASTED+CAFF compared with FASTED at the initial step of the test (Figure 3A).

RPE increased significantly step by step when the exercise intensity was enhanced (*P* < 0.001) (Figure 3B), but the change in RPE during the MFO rate test did not differ significantly between the experimental blocks (Figure 3B). Similarly, HR increased significantly step by step during the MFO rate test but did not differ significantly between experimental blocks at any intensity step (data not shown).

### Blood lactate and glucose concentration

Lactate changed significantly during the MFO rate test (*P* < 0.001). Lactate was lower after the first step of the MFO rate test (35% of PPO) than that in rest, but thereafter started accumulating and was significantly enhanced (3.3–4.2 mmol/L) at the last intensity step (72.5% of PPO). However, the change in lactate during the test did not differ between experimental blocks (*P* = 0.191) (Figure 3C).

Blood glucose was significantly different between experimental blocks during the MFO rate test (experimental block *P* < 0.001; experimental block × intensity interaction *P* = 0.04). Blood glucose during the MFO rate test in the FASTED+CAFF block was significantly higher than that on the FASTED block (mean: 5.1 ± 0.1 mmol/L compared with 4.7 ± 0.1 mmol/L; *P* < 0.001) and the FED block (mean: 4.8 ± 0.1 mmol/L; *P* = 0.016) (Figure 3D).

### 20TT test

**FIGURE 4:**
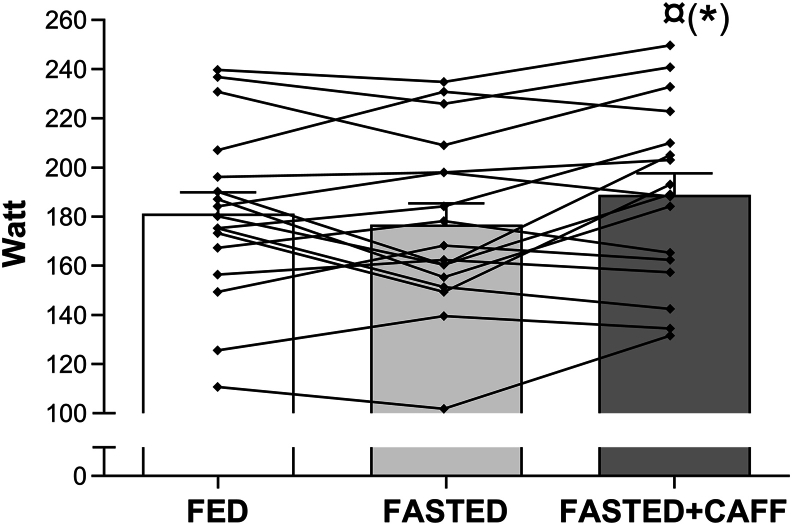
20-min time trial in the 3 experimental blocks. Data are shown as mean ± SEM (*N* = 17). Lines indicate individual power output (W) in each of the experimental blocks. ¤FASTED+CAFF > FASTED, *P* = 0.022; (∗) FASTED+CAFF > FED, *P* = 0.054. FASTED, no carbohydrate between exercise sessions; FASTED+CAFF, no carbohydrate between exercise sessions, but caffeine before exercise; FED, carbohydrate between exercise sessions.

The mean power output performed during the 20TT was significantly higher in FASTED+CAFF (189 ± 9 W; range: 131–250 W) than that in FASTED (177 ± 9 W; range: 101–235 W; mean: +6.9%; *P* = 0.022, effect size 0.32), and tended to be higher than that in FED (181 ± 9 W, range: 110–240; mean: +4.2%; *P* = 0.054, effect size 0.22) (Figure 4). The test for trial order effect did not influence the performance significantly (*P* = 1.0). Subjective reporting of motivation and sense of performance capacity before the physical test procedure (MFO rate and 20TT) did not differ significantly between the experimental blocks (Supplemental Figure 1) and was not influenced by test order.

### HIT session

On the first day of each experimental block, the HIT session was performed successfully. There were no differences in RPE and HR (% of max) between the experimental blocks (*P* = 0.220 and *P* = 0.198, respectively), but RPE rose significantly during the numbers of intervals (*P* < 0.001) (Table 2).

**TABLE 2:** HR and RPE during HIT.

	Interval 4	Interval 5	Interval 6	Interval 7
RPE				
FED	16.1 ± 0.4	16.6 ± 0.4	17.0 ± 0.3	17.6 ± 0.4
FASTED	15.6 ± 0.3	16.1 ± 0.3	17.0 ± 0.3	17.4 ± 0.4
FASTED+CAFF	15.3 ± 0.4	15.7 ± 0.4	16.4 ± 0.4	17.0 ± 0.3
Heart rate (% of max)				
FED	93.2 ± 1.0	93.6±0.9	93.8 ± 0.6	94.0 ± 0.6
FASTED	94.4 ± 0.7	94.7 ± 0.9	95.0 ± 0.7	95.0 ± 0.7
FASTED+CAFF	92.8 ± 1.0	93.2 ± 1.0	93.2 ± 1.0	93.9 ± 0.6

Values are mean ± SEM (*n* = 16).

*Abbreviations:* FASTED, no carbohydrate between exercise sessions; FASTED+CAFF, no carbohydrate between exercise sessions, but caffeine before exercise; FED, carbohydrate between exercise sessions; RPE, ratings of perceived exertion.

## Discussion

The main finding was that MFO rate was significantly higher in the FASTED+CAFF state than that in FED (small effect d=0.42), but not in the FASTED compared with that in FED state as hypothesized. Fat oxidation (g/min) was especially higher at the initial low intensity step of the MFO rate test in the FASTED+CAFF and FASTED period than that in the FED. Furthermore, as hypothesized, 20TT performance not only improved significantly by ∼7% in FASTED+CAFF compared with that in FASTED (small effect *d* = 0.32) but also tended to be improved compared with the FED situation (mean: +4.2%; *P* = 0.054, small effect *d* = 0.22) situation.

### FAT oxidation

Previous studies in trained males showed a higher fat oxidation rate in the FASTED than that in the FED state when applying a similar design [21] or after a 3-wk training period with manipulation of the endogenous and exogenous CHO availability during training sessions [11,12]. Nevertheless, although the fat oxidation curve in this study shifted upward and rightward from FED to FASTED condition, MFO rate was only significantly enhanced when the restriction of CHO in the recovery period after HIT was combined with caffeine supplementation in the morning before the MFO rate test, but not in the FASTED alone. No significant difference in fat oxidation rates and MFO rate was observed between the FASTED and FED condition in this trial contrasts with those by Lane et al. [21] even though the dietary plan and the design of the HIT protocol used in this study was adapted from that study. Sex differences and differential pre-exercise muscle glycogen stores may partly explain the discrepancy in the study results between the 2 studies. The HIT protocol used in Lane et al [21] and this study has previously been demonstrated to reduce muscle glycogen by ∼50% of starting values in well-trained male cyclists [27]. Likewise, Lane et al. [21] reduced muscle glycogen content with ∼50% in trained men but due to high starting values (∼600 mmol glycogen/kg dry weight), they observed a substantial amount of glycogen remaining in the muscles (>360 mmol/kg dry weight) both after the HIT session and in the morning before the training session. Regardless of prohibited CHO intake after HIT in the FASTED state, the athletes therefore commenced the next morning’s training session with glycogen availability higher than anticipated. Still, Lane et al. [21] observed significantly greater total fat oxidation in FASTED state than that in FED state. The females in this study were similar to the males in the study by Lane et al. [21], provided with 8 g CHO/kg before HIT on the FASTED day. Since FFM is both absolutely and relatively lower in females than males [28] the muscle glycogen content in the female athletes in this study has probably been at least as high as in the male participants in the study Lane et al [21]. Furthermore, the energy cost during HIT can be assumed to be lower in the females than that in males when using the same exercise and diet protocol due to the females exercise at a lower absolute workload in watt. Moreover, a study investigating gender differences in whole-body fat oxidation kinetics during exercise reported that females exhibited greater fat oxidation rates from 35% to 85% $\dot{V}$O_2max_ [29], indicating that the utilization of muscle glycogen during the HIT may have been lower in this study than that in the study by Lane et al [21]. In support of a lower utilization of muscle glycogen in females than that in males during a comparable HIT session, a study showed that females used 50% less muscle glycogen than males during a single bout of HIT [30]. Therefore, an explanation for not observing a marked difference in MFO rate during exercise in the morning in the FASTED block compared with that in the FED block may be related to the availability of muscle glycogen as fuel was not sufficiently lowered in the FASTED block to enhance the use of fat as fuel significantly compared with the control situation (FED). Unfortunately, no muscle biopsies were obtained to confirm this statement. The estimated effect size of combining CHO periodization with caffeine can be considered low-to-medium based on a Cohen *d* estimate of 0.44 [31]. Whether such an acute effect accumulates to beneficial adaptations in skeletal muscle and/or whole-body metabolism in endurance-trained females could therefore present an interesting question for future studies.

The fat oxidation levels during the MFO rate test in FASTED+CAFF condition appeared to be higher at lower intensities but lower at higher intensities than those during the FASTED condition. The mechanistic explanation for this observation cannot be determined from this study. However, higher lactate concentrations during exercise have been reported following coffee consumption than those during decaffeinated coffee or placebo [32], which may have influenced RER measurements by increasing carbon dioxide excretion from the bicarbonate pool, resulting in and thereby potentially leading to and underestimation of fat oxidation [23]. As shown in Figure 3, the lactate concentration in the blood was numerically, but not statistically, higher at the highest intensities in the FASTED+CAFF condition than those in the FASTED condition. However, further investigation is needed to determine whether caffeine influences fat oxidation rates and why.

In this study, a large interindividual variability in MFO rate ranging from 0.31 to 0.99 g/min was observed, being consistent with other studies [[33], [34], [35]] that observed interindividual variation in MFO rate tested in larger cohorts (range: 0.18–1.01 and 0.17–1.27 g/min, respectively). Goedecke et al. [36] concluded that training volume, dietary fat intake, muscle glycogen content, and circulating substrates accounted for 58%, 45%, 42%, and 56%, respectively, of the variability in RER as an indicator for substrate utilization in the skeletal muscle. The large interindividual variability suggests that effects of treatment (experimental block) on fat oxidation rates can only be detected if remarkable metabolic changes are induced and other confounding factors are eliminated by standardization. In this study with moderately endurance-trained females, only periodized CHO intake in combination with a caffeine supplement seemed to be potent enough to induce significant metabolic changes compared with the FED state.

Owing to the potential effect of menstrual cycle on substrate oxidation, the subjects in this study were during all 3 experimental blocks tested in the midluteal phase of their menstrual cycle to control for the effect of menstrual cycle phase on the utilization of fat. MFO rate in the FED (0.50 ± 0.04 g/min), FASTED (0.55 ± 0.04 g/min), and FASTED+CAFF state (0.57 ± 0.04 g/min) was higher than those reported in the study by Venables et al. [34] who in 143 females observed a mean MFO rate of 0.46 ± 0.01 g/min (range: 0.18–1.01 g/min). Similarly, Fat_max_ (∼66%–69% $\dot{V}$O_2max_; range 53%–86%) in this study was higher than that reported by Venables et al. [34] (48% ± 1% $\dot{V}$O_2max_; range: 25%–77%). The generally high whole-body fat oxidation rates during exercise in this study may, on top of the dietary manipulation of CHOs, be related to the females were tested in the luteal phase. Previous studies have reported greater whole-body fat oxidation during exercise in the midluteal phase of the menstrual cycle than that during the follicular phase [[37], [38], [39], [40]]. Additionally, sex differences in substrate utilization seem to be most distinct in the luteal phase [38,40], where the estradiol concentration is enhanced compared with the early and mid-follicular phase. Notably, Venables et al. [34] did not control for menstrual phase. If the females in Venables et al. [34] were tested primarily in the early-mid follicular phase, it could potentially have reduced the fat oxidation rates and thereby the MFO rate. The discrepancy between the studies may also be related to the females included in Venables et al. [34], who were at a lower fitness level ($\dot{V}$O_2max_: 41.4 ± 0.9 mL O_2_/min/kg) than the females in this study ($\dot{V}$O_2max_: 52.2 ± 1.2 mL O_2_/min/kg). This is supported by the study of Venables et al. [34], which showed that $\dot{V}$O_2max_ was a predictor of MFO rate (*r* = 0.26; *P* < 0.01). Furthermore, that endurance trained individuals use more fat at the same relative (higher absolute) exercise intensity than untrained individuals has been documented in several studies [24,33,[41], [42], [43], [44]].

A protein drink (0.4 g/kg BM protein) was included after HIT in all 3 experimental blocks to mimic practice in a real-life situation. Ingestion of protein after an exercise bout has been suggested to increase skeletal muscle protein synthesis and support muscle repair and remodeling [45,46]. Therefore, many athletes in a real-world setting ingest protein in the acute recovery phase to minimize skeletal muscle breakdown. However, the exogenous amino acids may have contributed to muscle glycogen resynthesis by providing substrate for gluconeogenesis during the recovery period between HIT and the morning exercise, even though the protein intake was relatively small [47]. This may potentially have enhanced the glycogen stores and thereby further counteracted that glycogen concentrations have been lowered to a degree that could significantly affect fat oxidation in the FASTED state.

### Performance

Caffeine supplementation enhanced the effect of restricting CHO intake in the recovery period after HIT on substrate utilization during the MFO rate test the following morning. Before the study, it was hypothesized that caffeine would enhance 20TT performance in a situation with comparable CHO availability. In line with this, a 7% higher performance in FASTED+CAFF was observed than that with both FASTED and FED. The observed ergogenic effect of caffeine supplementation is consistent with other time-trial studies in males, which have tested the effect of caffeine supplementation with comparable CHO availability in the test situations [25,48,49]. Interestingly, a tendency toward improved 20TT performance was observed in the FASTED+CAFF block compared with that in the FED block (*P* = 0.054), with no significant difference in 20TT performance between the FASTED and FED blocks. A reduced CHO availability has previously been coupled with reduced endurance work capacity [3]. As discussed earlier, the muscle glycogen has probably not been lowered sufficiently in this study in the FASTED block to have a marked negative impact on 20TT performance. Therefore, the convincing evidence of caffeine as ergogenic drug was able not only to counteract a numeric nonsignificant small decline in 20TT performance but to increase performance compared with the FED block.

There seems to be a consensus around caffeine’s performance enhancing effects through its stimulation of the central nervous system [50,51]. In a meta-analysis by Doherty and Smith [52], it was reported that caffeine dampened perceived exertion by 5.6% compared with placebo. Previously, it was suggested that caffeine enhanced fat utilization during exercise and thereby via a glycogen sparring effect was able to enhance endurance performance [53]. Nevertheless, later studies have not been able to document that the ergogenic effect of caffeine is coupled to enhanced fat oxidation and muscle glycogen sparing [[54], [55], [56]]. This study results open for future studies where the mechanistic ergogenic effect of caffeine is studied in females, especially in situation with reduced CHO availability. The observed higher blood glucose concentration in the FASTED+CAFF situation than that in FED may indicate that blood glucose was spared, and more fat was oxidized. This is consistent with a recent study by Lane et al. [17] who observed increased blood glucose concentration and augmented plasma free fatty acid concentration in trained males when 3 mg/kg caffeine was ingested 1 h before HIT with low glycogen availability compared with low glycogen availability and placebo [17]. However, future mechanistic studies are needed to explain the metabolic effect of caffeine supplementation on fat oxidation during exercise in females.

### Limitations

Owing to the experimental blocks were standardized according to the subjects’ menstrual cycle, 7 of the 17 subjects had >1 cycle between the experimental blocks due to illness. The median duration between blocks was 29 d (25%–75% were 28–47 d). Consequently, changes in training state over the course of the study could alter the relative intensity of the HIT sessions and MFO rate test and hence affect substrate utilization. However, the subjects’ individual monthly training history revealed no differences in training volume between the experimental blocks. Furthermore, BM and FM did not change significantly between the experimental blocks. Questionnaire data also indicate no change in sleeping pattern between the HIT session and the tests in the morning. Likewise, neither RPE nor HR differed between the experimental blocks when they performed the HIT sessions. These observations imply that the fitness level of the subjects and the load during testing did not change markedly throughout the study period. In addition, the use of a crossover design, with the order of FED, FASTED, and FASTED+CAFF balanced across subjects, minimizes the possibility that variations in the number of menstrual cycles, and thereby, months of training across experimental blocks could explain the results.

In the international society of sports nutrition position stand from 2023, it is concluded that a caffeine consumed in doses of 3–6 mg/kg improves exercise performance [57]. In this study, we used 100 mg pills. Therefore, for practical reasons, the dose of caffeine was not individualized per kilogram, but all participants received 3 pills corresponding to 300 mg caffeine. This absolute dose of caffeine corresponded to a range of 4.1–4.8 mg/kg, which means all participants received a dose within the recommended interval for experiencing an ergogenic effect. Habitual users of caffeine may experience side effects when suddenly refraining from consuming caffeine, but potential side effects in relation to the HIT session have likely been similar between blocks. Nevertheless, we cannot rule out that 20TT performance in the FED and FASTED state may have been negatively impacted by withdrawal of caffeine for ∼≥40 h in habitual users of caffeine/coffee consumers.

A limitation in this study is that no muscle biopsies were obtained and analyzed for muscle glycogen content. To achieve lower muscle glycogen content in the FASTED state before the tests on day 2 of the experimental block, the amount of CHO consumed before the HIT session in the FASTED state could have been reduced, thereby altering not only the timing but also the total CHO intake on day 1 of the experimental block. Furthermore, the difference between FED and FASTED block in MFO rate and 20TT had probably been greater if CHOs were served in the FED block before, during, and between the tests. Nevertheless, we chose to maintain the total energy and CHO intake similar in all blocks to isolate and study the effect of the periodization of the CHO intake around training sessions. Thereby, the CHO availability before the HIT training session at day 1 was lower in the FED block than in the FASTED blocks (5 compared with 8 g CHO/kg). Nevertheless, an intake of 5 g CHO/kg before the HIT session appeared sufficient to complete the HIT session in a satisfactory way, considering the participants the participants were physical inactive during day 1 before the HIT session.

## Conclusion

In conclusion, in endurance-trained female, fat oxidation rate at submaximal intensities and MFO rate during morning exercise is enhanced if the intake of CHOs in the recovery period between evening and morning exercise bouts is restricted and combined with caffeine supplementation before morning exercise. The effect of CHO restriction during recovery on fat oxidation is most pronounced at work intensities <65% of PPO. Caffeine supplementation before the exercise tests enhances 20TT performance when intake of CHO is restricted during recovery from a HIT session.

## Author contributions

The authors’ responsibilities were as follows—SR, MH: designed the study and obtained the funding the project; JFM, CS: conducted the experiments in the laboratory; CS: did the data analyses under supervision of SR, JFM, and MH; CS: wrote the first draft of the manuscript, which was thereafter edited by JFM, SR, and MH; and all authors: have read and approved the final version of the manuscript.

## Data availability

Data described in the manuscript, code book, and analytic code will be made available upon request pending application to the corresponding author and approval form The Danish Data Protection Agency.

## Funding

The research project was funded by Team Danmark. The protein powder was donated by Arla Foods Ingredients Group P/S.

## Conflict of interest

MH reports financial support provided by Team Danmark and protein powder donated by Arla Foods Ingredients Group P/S. The other authors report no conflicts of interest.

## Figures and Tables

**Figure:**